# Investigating the Role of Peroxisomes in Regulating Breast Cancer Stem Cell Mechanisms

**DOI:** 10.3390/ijms262311389

**Published:** 2025-11-25

**Authors:** Deniz Simsek, Ghada S. Hassan, Federica Sotgia, Michael P. Lisanti

**Affiliations:** 1 Translational Medicine, School of Science, Engineering and the Environment (SEE), University of Salford, Greater Manchester M5 4WT, UK; denizsimsek585@gmail.com; 2 Department of Basic Pharmaceutical Sciences, Faculty of Pharmacy, Hacettepe University, Ankara 06230, Turkey; 3 Lunella Biotech, Ottawa, ON K1Z 7K4, Canada; ghada.s.hassan@gmail.com; 4 The Institute of Mental Health Research (IMHR), University of Ottawa, The Royal Ottawa Hospital (ROH), 1145 Carling Ave, Ottawa, ON K1Z 7K4, Canada

**Keywords:** peroxisomes, MCF-7 cells, cancer, stemness, PPARγ, ROS, ferroptosis

## Abstract

Cancer and ageing remain major challenges for humanity, requiring innovative solutions. While the role of mitochondria in cancer and ageing has been extensively studied, peroxisomes have received comparatively little attention in this context. In our study, we investigated the impact of peroxisomes on cancer stemness. We systematically analysed the metabolic differences between MCF-7 cells with low- and high-peroxisome levels. Briefly, MCF-7 cells were stably transduced with GFP- and RFP-fluorescent reporters that were targeted to peroxisomes, by addition of a C-terminal SKL (Serine-Lysine-Leucine) peroxisomal targeting signal. To independently validate our observations, MCF-7 cells were also treated with Rosiglitazone, a peroxisome proliferator-activated receptor gamma (PPARγ) agonist that enhances peroxisome levels. Key parameters examined included cancer stemness, levels of reactive oxygen species (ROS), cell division dynamics, autophagy activity, the DNA damage response, susceptibility to ferroptosis, mitochondrial respiration, and aerobic glycolysis in cells with low- and high-peroxisome profiles. Our results demonstrated that elevated peroxisome levels significantly decrease the capacity of breast cancer stem cells (BCSCs) to form mammospheres or colonies, thus reducing their stemness potential. In high-peroxisome cells, Mammosphere formation was reduced by approximately 50%, and colony formation by 80% compared to low-peroxisome cells. This decline in stemness was accompanied by an approximately one-and-a-half-fold increase in ROS levels and a five-fold increase in lipid peroxidation, reflecting increased mitochondrial lipid peroxidation and ferroptosis. Continued research is, however, essential to further validate these findings and to elucidate the underlying mechanisms.

## 1. Introduction

Peroxisomes are single-membrane organelles present in most eukaryotic cells, containing a variety of enzymes crucial for essential metabolic processes, including lipid metabolism and reactive oxygen species (ROS) homeostasis [1]. Alongside mitochondria, peroxisomes serve as significant sources of ROS. However, peroxisomes contain the antioxidant enzyme catalase, which efficiently neutralises ROS and prevents oxidative damage [2]. Given the critical role of peroxisomes in maintaining metabolic homeostasis, any dysfunction in these organelles can lead to significant physiological impairments, including disrupted redox balance, impaired lipid metabolism, mitochondrial dysfunction, altered gene expression, increased endoplasmic reticulum (ER) stress, and ultimately cell death [3].

Cancer and ageing are major health concerns worldwide. Even though cancers can occur at any age, the risk of developing tumours rises in older people. Ageing and cancer are closely interconnected. They are both characterised by some common processes, such as the increased incidence of mutation and accumulation of mutated cells [4], loss of epigenetic changes that define cellular identities [5], upregulated inflammatory responses [6,7] and dysbiosis [8,9]. Both processes are also influenced by metabolic reprogramming, involving disturbances at the level of mitochondria as well as peroxisomes as organisms age. Breast cancer remains the most frequently diagnosed cancer and the leading cause of cancer-related death among females worldwide. In 2022, it ranked as the first or second most common cancer in 183 of 185 countries, and projections estimate that by 2050, global cases and deaths will rise by approximately 38% and 68%, respectively [10]. Therefore, investigating the underlying mechanisms and developing effective therapeutic strategies for breast cancer are of critical importance.

Mitochondria have long been recognised as a major factor in the ageing and tumorigenesis processes [11,12]. Similarly to mitochondrial dysfunction, the disruption at the level of peroxisomes leads to oxidative damage, contributing to the ageing process and the development of cancer [13]. In fact, mitochondria and peroxisomes are now known to be closely interacting organelles involved in several cellular processes, including lipid metabolism, ROS signalling, and protein exchange [14,15]. Their interaction is of particular importance in the context of ROS metabolism and maintaining oxidative balance within the cell [14,15]. It was shown that any disruption in peroxisomal ROS clearance mechanisms, or inhibition of peroxisomal catalase, can have a detrimental effect on mitochondrial shape and function [14,16]. Indeed, localised oxidative damage to peroxisomes, combined with increased intraperoxisomal ROS production, can result in mitochondrial fragmentation, potentially leading to cell death [14]. This highlights the importance of balanced ROS regulation between the two organelles to ensure cellular health and longevity, hence their role in processes related to ageing and cancer.

While the role of mitochondria in cancer and ageing is well established [17] the significance and impact of peroxisomes in these processes have been largely overlooked and remain poorly understood. In addition to catalase levels declining in aged rodent and human cells, catalase deficiency has been shown to accelerate the ageing process in *C. elegans* [14]. Several studies have also shown that increasing catalase activity can protect organisms from oxidative stress and promote longevity [18,19,20,21]. This highlights the role of peroxisomes and their enzymes in protecting against oxidative damage and its potential impact on longevity.

As to cancers, studies have revealed that peroxisome abundance and activity can alter greatly across different cancer types and their specific microenvironments, underscoring the functional role of peroxisomes in tumorigenesis. In certain cancers, peroxisome levels and functions are upregulated, supporting cancer cell survival and growth, while in other cancers, these levels are diminished, potentially reflecting distinct metabolic demands [1]. This dynamic behaviour suggests that peroxisomes play a context-dependent role in cancer progression. In a lymphoma model, it has been demonstrated that peroxisome biogenesis factors, such as PEX3, PEX16, and PEX19, contribute to tumorigenesis [22]. Specifically, increased peroxisome levels are associated with disease progression. Notably, silencing PEX3 to inhibit peroxisome function resulted in a 70% increase in cell death, suggesting that reducing peroxisome levels decreases the resistance of lymphoma cells to treatment. In contrast, levels of peroxisomes and the activity of their key enzyme, catalase, as well as of two enzymes involved in the peroxisomal β-oxidation pathway, were shown to be significantly reduced in colon carcinoma [23]. Furthermore, treatment with troglitazone, a peroxisome proliferator-activated receptor gamma (PPARγ) ligand, significantly diminished colon cancer growth in mice [24]. In addition to colon cancer, catalase activity was decreased in breast cancer patients [25]. Moreover, patients who died from the disease exhibited markedly lower PPARγ expression, highlighting its association with poor prognosis. In line with this, PPARγ levels have been reported to be significantly reduced in metastatic breast cancer tissues compared to primary tumours [26]. Similarly, the highly metastatic breast cancer cell line MDA-MB-231 exhibits lower PPARγ expression compared to the non-metastatic MCF-7 cells [27]. These latter findings suggest that low levels of PPARγ, peroxisomes, and catalase in cancer cells may contribute to tumour progression, and increasing those levels can potentially inhibit cancer growth.

Given the observed link between peroxisomes, cancer, and ageing, there is growing interest in targeting peroxisomal pathways as potential therapeutic strategies for both cancer treatment and age-related diseases. Therefore, in this study, we generated two populations of breast cancer cells, MCF-7 cells, with high- or low-peroxisome levels. We aimed to explore how peroxisome levels affect cancer stemness and to understand the relationship between peroxisomes, cancer, and ageing, with the aim of identifying novel therapeutic approaches that address both conditions.

## 2. Results

### 2.1. Generation and Sorting of High- and Low-Peroxisome Cells

**Figure 1:**
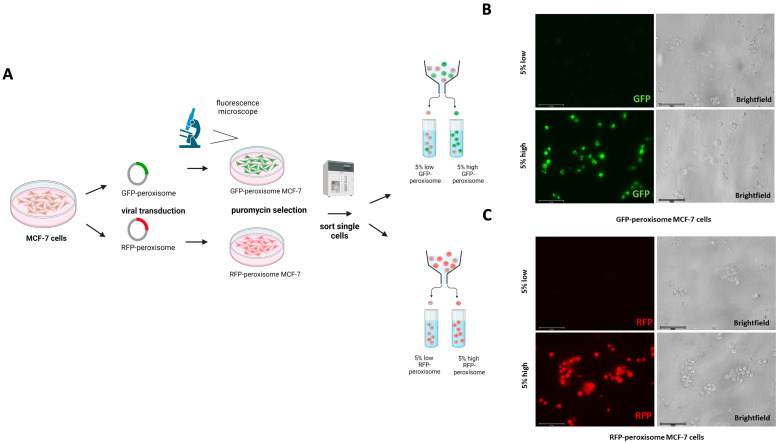
Generation and sorting of GFP-peroxisome- and RFP-peroxisome-tagged MCF-7 Cells. (**A**) A schematic representation of the viral transduction and sorting process was shown. MCF-7 cells were transduced with viral vectors carrying GFP-peroxisome and RFP-peroxisome tags. Following transduction, cells were sorted based on fluorescence intensity into 5% low and 5% high populations for both GFP-peroxisome and RFP-peroxisome. (**B**) Representative fluorescence images of sorted GFP-peroxisome cells showing the fluorescence intensity of GFP-tagged peroxisomes in the 5% low and 5% high populations. (**C**) Representative fluorescence images of sorted RFP-peroxisome cells displaying the fluorescence intensity of RFP-tagged peroxisomes in the 5% low and 5% high populations. Schematics were created using BioRender.

In this study, we investigated the influence of peroxisomes on the progression of cells into a cancerous phenotype. Specifically, we focused on MCF-7 cells with higher levels of peroxisomes to investigate their role in cancer stem cells (CSCs) metabolism. To achieve this, we transduced the MCF-7 cell line with two lentiviral constructs that label peroxisomes at the sub-cellular level, each under the control of GFP or RFP expression. This allowed us to differentiate between lower and higher peroxisome populations using flow cytometry. Subsequently, stably transduced cells were processed through flow cytometry to isolate the 5% highest GFP (high GFP-peroxisome) and lowest 5% GFP (low GFP-peroxisome) subpopulations, as well as the 5% highest RFP (high RFP-peroxisome) and lowest 5% RFP (low RFP-peroxisome) subpopulations (Figure 1A). After cell sorting, fluorescence microscopy confirmed the successful isolation of GFP low and GFP high sub-populations (Figure 1B), as well as RFP low and RFP high sub-populations, distinguishing two distinct populations based on fluorescence intensity (Figure 1C).

### 2.2. High-Peroxisome MCF-7 Exhibit Reduced Mammosphere and Colony Formation as Compared to Low-Peroxisome Cells

**Figure 2:**
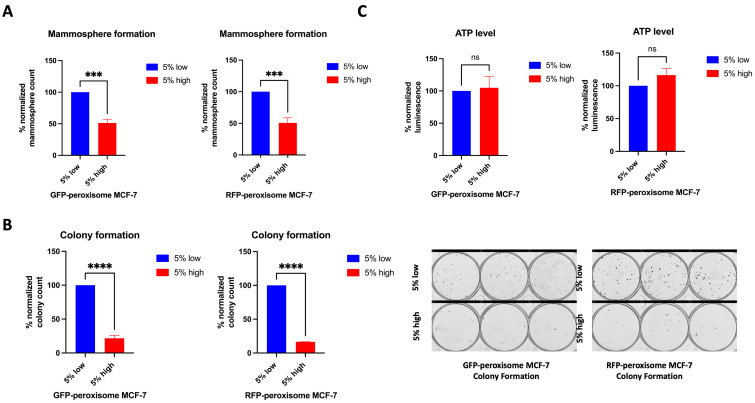
Impact of peroxisome levels on Mammosphere and colony formation, and ATP levels. High-peroxisome level is associated with decreased Mammosphere formation and colony-forming ability of MCF-7 cells, while ATP levels between the high-peroxisome and low-peroxisome cells remained unchanged. (**A**) Cells were seeded on Poly-HEMA-coated wells in Mammosphere media and incubated undisturbed for 7 days. Mammospheres were then counted (N = 3, *n* = 3). (**B**) Cells were seeded as a single cell in 10% DMEM and incubated undisturbed for 14 days. Formed colonies were fixed with 70% ethanol, stained with 0.5% crystal violet, and then counted using a GelCount (N = 3, *n* = 3). (**C**) The sorted low- and high-peroxisome cells were incubated with Promega CellTiter-Glo Luminescent Cell Viability Assay (G7570) for 15 min, and the luminescence level indicating ATP amount in the cell was read out (N = 3, *n* = 3). Bar graphs are shown as the mean ± SD; unpaired *t*-test. *** = *p* < 0.001; **** = *p* < 0.0001.

We studied the role of peroxisomes in the propagation of breast cancer stem cells (BCSCs) by comparing the Mammosphere formation ability between the top 5% highest and bottom 5% lowest subpopulations of GFP-peroxisome and RFP-peroxisome MCF-7 cells. Peroxisome-high populations exhibited a significant decrease in Mammosphere formation, which functionally represents anchorage-independent BCSCs propagation under 3D conditions (Figure 2A). Further investigating the effect of peroxisomes on cell proliferation behaviour, we compared the colony formation ability between these subpopulations. Peroxisome-high cells showed a significant decrease in colony formation, indicating the impact of peroxisomes on cell survival and proliferation (Figure 2B). To further elucidate the role of peroxisomes in BCSCs’ behaviour, we analysed the metabolic profiles of cells, particularly ATP production. Typically, ATP levels and Mammosphere and colony formation are expected to correlate, reflecting cancer stemness. Since cells with lower peroxisome content are associated with higher stemness, their metabolism—and consequently their ATP level—was anticipated to be higher than that of cells with higher peroxisome content. However, we did not observe any significant difference in ATP levels between the high- and low-peroxisome populations (Figure 2C).

These data suggest that cancer stemness decreases with higher peroxisome levels, despite similar metabolic activity between high- and low-peroxisome cell populations, as ATP levels remained unchanged in both groups.

### 2.3. High-Peroxisome MCF-7 Cells Demonstrate Higher Upregulation of Oxidative Stress than Low-Peroxisome Cells

**Figure 3:**
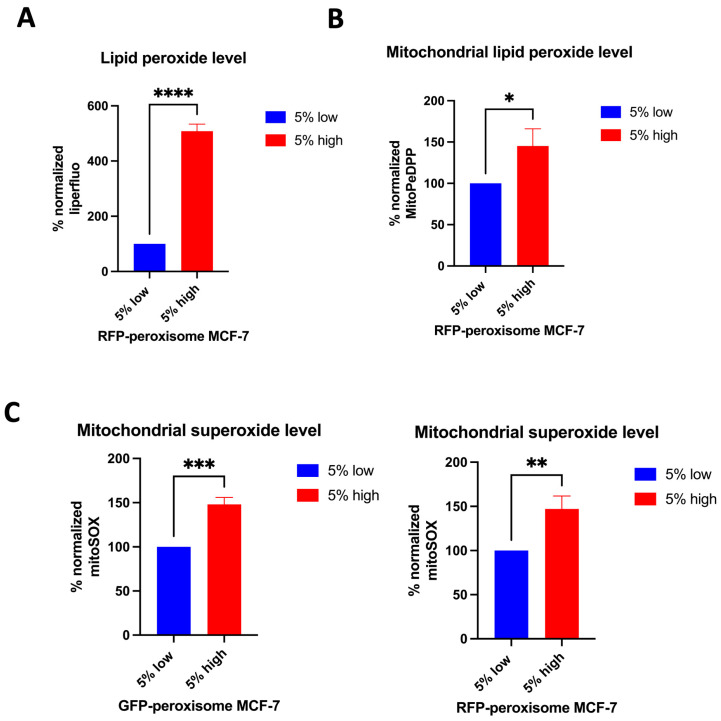
The relationship between peroxisome abundance and various markers of oxidative stress in MCF-7 cells. Cells with high levels of peroxisomes exhibit elevated levels of lipid peroxide, mitochondrial lipid peroxide, and mitochondrial superoxide compared to cells with low levels of peroxisomes. (**A**) Lipid peroxide levels were obtained by staining cells with Liperfluo (N = 3, *n* = 4). (**B**) The mitochondrial lipid peroxide levels were assessed by staining the cells with MitoPeDPP (N = 3, *n* = 2). (**C**) The level of mitochondrial superoxide was determined by staining the cells with MitSOX Deep Red (N = 3, *n* = 2). Stains were analysed using flow cytometry. Bar graphs are shown as the mean ± SD; unpaired *t*-test. * = *p* < 0.05; ** = *p* < 0.01; *** = *p* < 0.001; **** = *p* < 0.0001.

We further investigated the effect of peroxisomes on key oxidative stress parameters. First, the level of lipid peroxide in high- and in low-peroxisome cells was evaluated using the Liperfluo probe and flow cytometry [28]. High-peroxisome subpopulation demonstrated a significant increase in lipid peroxide levels (Figure 3A). Next, we determined levels of mitochondrial lipid peroxide in the high- and low-peroxisome populations by MitoPeDPP [29]. Our data showed that mitochondrial lipid peroxide is more upregulated in the high-peroxisome cells than in the low-peroxisome ones (Figure 3B). As to the mitochondrial superoxide level, assessed using the MitSOX probe [30], it was significantly higher in the high-peroxisome cells compared to the low-peroxisome group (Figure 3C). These findings suggest a potential link between peroxisome abundance and oxidative stress, indicating that higher peroxisome levels may contribute to increased oxidative damage within the cell.

### 2.4. High-Peroxisome Cells Show Higher Autophagy, DNA Damage, and Intracellular Fe^+2^ Levels than Low-Peroxisome Cells

**Figure 4:**
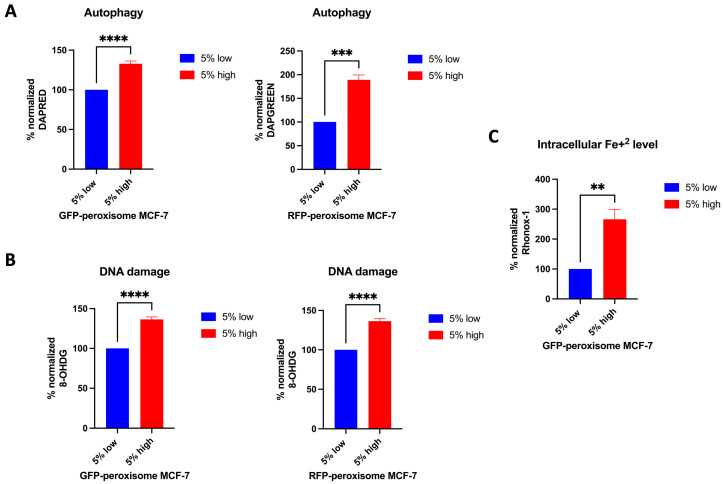
Correlation between peroxisome abundance and cellular processes such as autophagy, DNA damage, and ferroptosis. Autophagy, DNA damage, and intracellular Fe^+2^ levels were higher in cells with high-peroxisome levels as compared to low-peroxisome cells. (**A**) Autophagy levels in GFP-peroxisome and RFP-peroxisome cells were assessed by staining the cells with DAPRED and DAPGREEN probes, respectively, followed by analysis through flow cytometry (N = 3, *n* = 3). (**B**) DNA damage levels were measured by probing cells with an 8-OHdG conjugated Alexa Fluor 647 antibody, followed by analysis via flow cytometry (N = 3, *n* = 2). (**C**) Intracellular Fe^2+^ levels were determined by staining the cells with RhoNox-1 and analysing them via flow cytometry (N = 3, *n* = 2). Bar graphs are shown as the mean ± SD; unpaired *t*-test. ** = *p* < 0.01; *** = *p* < 0.001; **** = *p* < 0.0001.

Next, we investigated the impact of peroxisomes on additional cellular processes, including autophagy, DNA damage, and ferroptosis. For autophagy assessment, GFP-peroxisome MCF-7 cells and RFP-peroxisome MCF-7 cells were incubated with DAPRED and DAPGREEN probes, respectively, and analysed using flow cytometry. Our data showed that high-peroxisome cells exhibited a significantly higher rate of autophagy compared to low-peroxisome cells (Figure 4A). In addition, we evaluated DNA damage in these populations of cells by assessing 8-OHdG, an oxidised derivative of deoxyguanosine and indicative of oxidative damage of DNA [31]. Consistent with the autophagy results, DNA damage was significantly elevated in high-peroxisome cells compared to low-peroxisome cells (Figure 4B). As for ferroptosis, a form of regulated cell death associated with oxidative damage, different from apoptosis [32] and driven primarily by iron accumulation, lipid peroxidation, and the resulting disruption of the plasma membrane [33], it was assessed herein by measuring intracellular Fe^2+^ levels. Cells were incubated with the Rhonox-1 probe and analysed via flow cytometry. In line with the results of lipid peroxide levels and autophagy reported above, intracellular Fe^2+^ levels were significantly higher in high-peroxisome cells compared to low-peroxisome cells (Figure 4C). These findings suggest a potential correlation between peroxisome abundance and key cellular processes, such as autophagy, DNA damage, and iron homeostasis.

### 2.5. High-Peroxisome Cells Exhibit Similar Cell Cycle Distribution to That of Low-Peroxisome Cells

**Figure 5:**
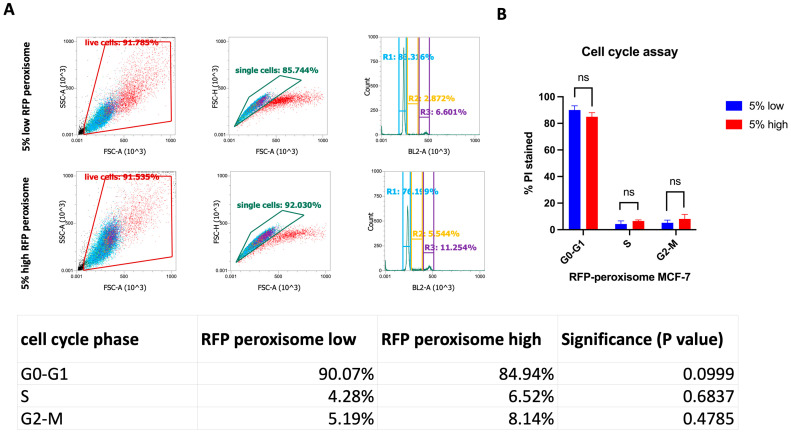
Assessment of cell distribution in the various cell cycle phases between high- and low-peroxisome cells. There are no significant differences in the cell cycle phases (G0-G1, S, G2-M) between low-peroxisome and high-peroxisome cells. (**A**) Flow cytometry charts showing the distribution of live, single, and cell cycle phases between the two populations are shown as a representative example. (**B**) Cell distribution is assessed using propidium iodide, which binds to DNA and reflects the DNA content of cells. Data are represented as % of PI-positive cells in each cell cycle phase. Bar graph is shown as the mean ± SD; unpaired *t*-test (N = 3, *n* = 1).

We further investigated the potential influence of peroxisomes on cell growth and division. To assess this, high- and low-RFP-tagged-peroxisome cells were stained using the Muse^®^ Cell Cycle Kit, a propidium iodide (PI)-dependent assessment of DNA content that measures the distribution of cells in the various cell cycle phases. Flow cytometry results (Figure 5A) indicated no significant differences between the high- and low-peroxisome groups. The proportion of PI-stained cells was similar across the G0-G1, S, and G2-M phases in both cell populations (Figure 5B). Statistical analysis (Figure 5) confirmed the absence of significant differences between the high- and low-peroxisome cells in terms of cell cycle phase distribution.

### 2.6. High- and Low-Peroxisome Cells Show Similar Mitochondrial Respiration and Glycolytic Function

**Figure 6:**
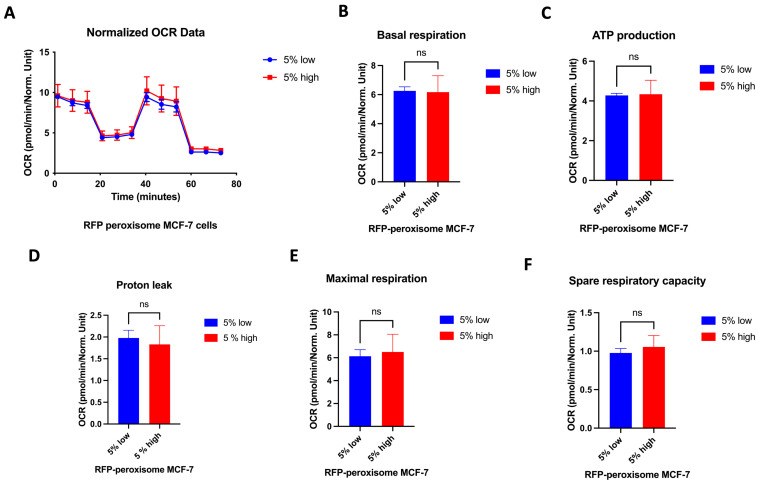
Influence of peroxisome levels on mitochondrial respiration. High- and low-peroxisome cells show no difference in terms of mitochondrial respiration. Mitochondrial respiration was assessed by measuring the OCR using the Seahorse XFe96 metabolic-flux analyser in low- and high-peroxisome cells. (**A**) The kinetic graph represents the whole mitochondrial respiration following sequential addition of inhibitors, and from which are derived the following panels: basal respiration (**B**), ATP-linked respiration and respiration of proton leak both assessed following addition of oligomycin (**C**,**D**), maximal respiration following injection of carbonyl cyanide-p-trifluoromethoxy phenylhydrazone (FCCP) (**E**), and residual respiration assessed following addition of a combination of rotenone and antimycin A (**F**). Bar graphs are shown as the mean ± SD; unpaired *t*-test (N = 3, *n* = 3).

**Figure 7:**
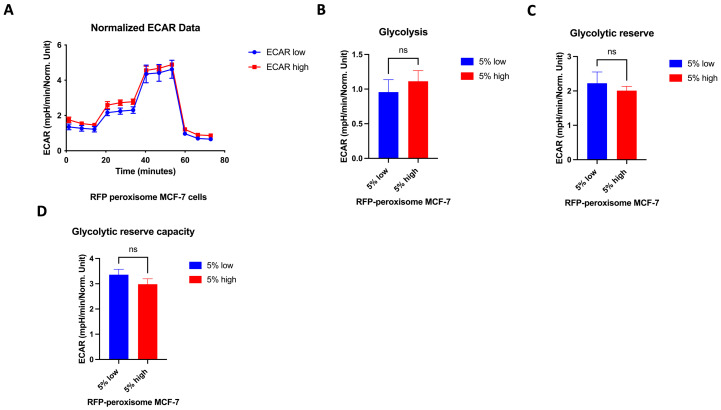
Effect of peroxisome level on glycolytic function. Aerobic glycolysis is similar in cells with high- and low-peroxisome levels. The glycolytic function was assessed by measuring the ECAR using the Seahorse XFe96 metabolic-flux analyser in high- and low-peroxisome populations. (**A**) The kinetic graph represents the glycolytic function following the addition of glucose, oligomycin, and 2-deoxyglucose (2-DG), and from which the subsequent panels are derived: basal glycolysis evaluated upon addition of glucose (**B**), the glycolytic capacity assessed following the addition of oligomycin (**C**), and the glycolytic reserve upon addition of 2-DG (**D**). Bar graphs are shown as the mean ± SD; unpaired *t*-test (N = 3, *n* = 3).

It is well established that the proliferation of CSCs relies, at least in part, on enhanced mitochondrial biogenesis and metabolic activity [34]. We assessed a possible effect of peroxisomes on metabolic functionality of the cell by measuring oxygen consumption rate (OCR) and extracellular acidification rate (ECAR), using the Seahorse XFe96 bioenergetic analyser. Our data showed that high- and low-RFP-peroxisome cells exhibit a similar metabolic profile. There is almost no difference between the cell populations in terms of mitochondrial respiration (Figure 6A–F) as well as glycolytic function (Figure 7A–D).

### 2.7. Cells Treated with a PPARγ Agonist Exhibit a Stemness Profile Similar to That of High Peroxisome Cells

**Figure 8:**
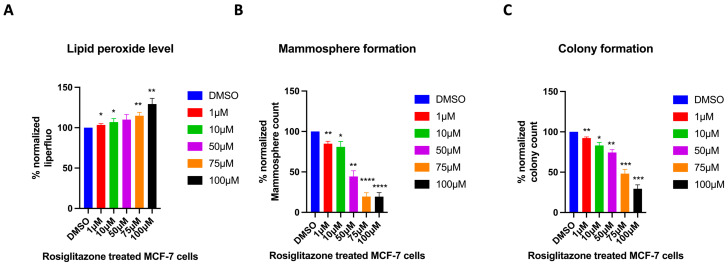
Evaluating the effect of the PPARγ agonist, Rosiglitazone, on MCF-7 cells. Treating cells with the PPARγ agonist Rosiglitazone mimics the high-peroxisome cell profile. (**A**) Lipid peroxide levels were obtained by staining cells with Liperfluo and analysed by flow cytometry (N = 3, *n* = 3). (**B**) Cells were seeded on Poly-HEMA-coated wells in Mammosphere media and incubated undisturbed for 7 days. Mammospheres were then counted (N = 3, *n* = 3). (**C**) Cells were seeded as a single cell in 10% DMEM and incubated undisturbed for 14 days. Formed colonies were fixed with 70% ethanol, stained with 0.5% crystal violet, and then counted using a GelCount (N = 3, *n* = 3). Bar graphs are shown as the mean ± SD; unpaired *t*-test. * = *p* < 0.05; ** = *p* < 0.01; *** = *p* < 0.001; **** = *p* < 0.0001.

Among the regulators of peroxisome function is PPAR [35]. PPARγ is part of the nuclear hormone receptor superfamily, which has a central role in regulating adipocyte differentiation, glucose, and lipid metabolism [36]. Rosiglitazone, a PPARγ agonist, has been used clinically for the treatment of type 2 diabetes mellitus (T2DM) [37]. Here, we aim to evaluate whether the effect of Rosiglitazone on cells would mimic the high-peroxisome profile described above, in terms of cancer stemness characteristics. Our data show that treating wild-type MCF-7 cells with increasing concentrations of Rosiglitazone significantly increased their lipid peroxide levels (Figure 8A). This result suggests that Rosiglitazone-treated MCF-7 cells exhibit a similar lipid peroxidation profile to that of high-peroxisome MCF-7 cells. Subsequently, we evaluated the effect of Rosiglitazone on Mammosphere and colony formation in wild-type MCF-7 cells. Mammosphere formation decreased significantly with higher concentrations of Rosiglitazone (Figure 8B), mirroring the reduced Mammosphere formation observed in high-peroxisome MCF-7 cells. Treatment with increasing Rosiglitazone concentrations led to a reduction in colony numbers in wild-type MCF-7 cells (Figure 8C), as was the profile of high-peroxisome cells. Altogether, these data highly suggest that Rosiglitazone, a PPARγ agonist and a known peroxisome proliferator, as previously discussed [37], could be used to enhance peroxisome activity in MCF-7 breast cancer cells and potentially reduce their stemness profile.

## 3. Discussion

Cancer and ageing are closely linked and remain major challenges in need of innovative solutions and effective treatments. Gaining a deeper understanding of the pathways and identifying new indicators and mechanisms involved in cancer and ageing is essential for advancing effective interventions. When it comes to cancer and ageing-related diseases, peroxisomes—often underestimated organelles—might play a crucial role, alongside mitochondria, in regulating both cancer progression and ageing processes. In this research, we investigated how peroxisomes influence these mechanisms specifically in breast cancer, potentially providing new approaches for treatment and intervention in cancer and ageing in the future. To achieve this, we re-engineered the MCF-7 cell line by tagging peroxisomes with either GFP or RFP, sorted cells with low- and high-peroxisome levels, and evaluated their stemness potential and metabolic activity.

Stemness is the ability of the cell to self-renew. Normal stem cells exhibit stemness to maintain homeostasis, while cancer cells show stemness when involved in malignant growth [38]. With cancer progression, cell differentiation is lost, and the tumour-initiating feature takes over in cells, resulting in their stem-like phenotype with the ability for self-renewal, unlimited propagation, and multipotent differentiation. These cells are called CSCs, believed to be the source of the tumour and also responsible for tumour progression, metastasis, resistance to therapy, and recurrence [39]. Therefore, targeting CSCs and identifying strategies to eliminate them is a potential strategy for cancer treatment. Peroxisomes, organelles responsible for numerous enzymatic reactions involved in maintaining cellular homeostasis and proper cellular functioning, are increasingly being investigated in the context of tumorigenesis. With the metabolic reprogramming signature of cancer cells, disturbances in their content of peroxisomes and their enzymes are highly expected. Different levels and activities of peroxisomes have been demonstrated in different types of cancers. Some studies have revealed elevated levels of peroxisomal enzymes in some cancers [40,41,42]. Nevertheless, numerous tumours are characterised by decreased levels and/or function of these organelles. Human colon carcinoma cells were shown to exhibit a decreased or even absence of expression of some peroxisomal proteins, such as catalase and acyl-CoA oxidase enzymes, and some membrane proteins [23]. Another study demonstrated that samples from human breast and colon carcinomas, while maintaining peroxisome levels, showed decreased specific activity of peroxisomal enzymes [43]. Peroxisomes, but not their enzymatic activity (peroxisomal enzymes), were also revealed to be reduced in immunohistochemically stained sections of human hepatocellular carcinomas [44]. These findings support the hypothesis that upregulating these organelles and their activity might alter the stemness characteristic of these tumours. In our present study, we demonstrated that MCF-7 cells with high-peroxisome levels produced fewer mammospheres and formed fewer colonies as compared to low-peroxisome cells. This suggests that peroxisome abundance is associated with lower cancer stemness and that peroxisomes might have an anti-tumorigenic effect in breast cancer cells.

Interestingly, high cancer stemness is often associated with elevated ATP levels, as ATP is a key driver of aggressive cancer phenotypes [34,45]. As such, ATP levels can serve as a biomarker to detect and isolate aggressive CSCs’ characteristics [46]. However, our results revealed no notable alterations in ATP levels between high- and low-peroxisome cells, despite the significant differences in their cancer stemness. This suggests that the regulation of ATP levels and cellular metabolism in high-peroxisome cells might involve a different mechanism, leading to stable ATP levels under varying stemness conditions [34].

The metabolic characteristics of CSCs remain unclear, with conflicting evidence regarding their energy sources. Some studies suggest CSCs are primarily glycolytic, while others indicate they rely on mitochondrial respiration [34,47]. In this study, while high- and low-peroxisome cells had distinct stemness profiles, they demonstrated similar mitochondrial respiration rates, glycolysis levels, and division/growth potential. This suggests that while peroxisome levels influence stemness, they do not directly affect glycolysis, oxidative respiration, or cell growth. The lack of major metabolic changes suggests that peroxisomal β-oxidation may act as a compensatory mechanism to maintain cellular energy balance despite variations in peroxisome abundance [48]. This compensation likely allows cells to keep their basic energy state stable, while peroxisomal activity mainly influences redox balance and stemness-related signalling rather than directly changing metabolic flux. The absence of metabolic or proliferative differences, despite the clear divergence in stemness, underlines the complexity of CSC metabolism and the theory previously described of the metabolic adaptability of CSCs in response to their microenvironment [34].

ROS play a dual role in cancer biology, influencing both disease progression and regression. On one hand, ROS contribute to carcinogenesis by inducing DNA damage, disrupting signalling pathways, altering gene expression, and promoting cancer stemness. On the other hand, ROS have beneficial potential as they can induce apoptosis and autophagy in cancer cells [49]. In line with the latter effect of ROS, our data showed that low-peroxisome cells exhibiting increased stemness showed a reduced level of oxidative factors (lipid peroxide, mitochondrial lipid peroxide, and superoxide), probably underlying their decreased autophagy, DNA damage, and ferroptosis. Given these findings linking ROS production to apoptotic behaviour of tumour cells, the use of external agents to further increase ROS levels could selectively and efficiently target these cells. Recently, a novel therapeutic strategy has emerged that uses ROS-induced autophagy as a targeted approach to eliminate cancer cells [49,50]. Certain available anti-cancer drugs regulate ROS production through various mechanisms and have been used effectively to treat various types of cancer. As an example, Bauer et al. demonstrated that Vemurafenib, a drug used to treat melanoma, increases ROS production by causing depolarisation of the mitochondrial membrane in BRAF-mutated melanoma cells [51]. Another study showed that doxorubicin, a chemotherapy drug used to treat breast cancer, Kaposi’s sarcoma, bladder cancer, and acute lymphoblastic leukaemia, promotes increased ROS production through Fenton’s reaction and electron leakage [52]. Moreover, Gamitrinib, a drug used in the treatment of prostate cancer, enhances ROS production by inducing mitochondrial collapse [53]. Interestingly, our study demonstrates that high-peroxisome MCF-7 cells produce more ROS than low-peroxisome MCF-7 cells and are thus more prone to autophagy, DNA damage, and ferroptosis, which highly suggests that increasing peroxisomes in BCSCs might be an efficient therapeutic strategy to reduce tumorigenesis and treat breast cancer.

Peroxisomes and mitochondria are intimately linked through redox and metabolic communication, and both contribute to cellular ROS production [54,55]. Peroxisome-derived ROS can act as signalling molecules that influence mitochondrial dynamics and mitophagy, thereby modulating overall cellular homeostasis [56]. Excess peroxisomal ROS may activate apoptotic pathways that promote the selective removal of damaged mitochondria, while impaired peroxisomal function can disrupt this balance and exacerbate oxidative damage [57]. Thus, the observed increase in ROS following peroxisome elevation may reflect an adaptive signalling mechanism coordinating mitochondrial quality control rather than solely oxidative injury.

The link between low ROS production and cancer cell stemness highlighted in our study has been previously demonstrated [49,50]. Indeed, CSCs are known to have lower intracellular ROS levels compared to non-CSCs, and such low ROS production is linked to stemness [49,50]. One possible mechanism behind the low ROS levels in CSCs is the upregulation of free radical scavenging systems in these cells, which may contribute to their resistance to anti-cancer therapies [58]. By enhancing free radical scavenging systems, CSCs can shield themselves from oxidative stress, supporting their survival, self-renewal, and resistance to treatment. Therefore, increasing ROS levels in cancer cells has been explored as a treatment strategy in several studies to reduce their resistance to anti-cancer treatment. For example, menadione, a known ROS inducer, has been utilised for this purpose [59,60,61]. Treatment with menadione enhances ROS production in cancer cells, which lowers their resistance to anti-cancer therapies, ultimately improving anti-tumour efficacy [62]. These findings align with our results, suggesting that high-peroxisome levels, which lead to increased ROS production, could serve as an effective strategy for eliminating CSCs in breast cancer.

PPARs are nuclear receptors known to enhance peroxisome levels and function [36]. When activated by their ligands, these transcription factors regulate genes involved in peroxisome formation and enzymatic activity, and thus energy metabolism [63]. Given our interesting data showing the association of high peroxisomal content with decreased stemness, we sought to evaluate the role of a PPAR agonist that increases peroxisome biogenesis and function on CSCs’ stemness. As a PPAR-γ agonist, we chose Rosiglitazone, an FDA-approved drug for the treatment of T2DM, which enhances the sensitivity of cells to insulin [64]. Our findings underscoring the anti-stemness effect of Rosiglitazone and that the phenotype of Rosiglitazone-treated cells mirrors that observed in high-peroxisome/low stemness cells, highly suggest the use of this drug as a strategy to influence the stemness potential of cancer cells. In this study, Rosiglitazone was employed as an experimental tool to activate PPARγ and investigate its mechanistic role in peroxisome-mediated regulation of stemness as explained above. However, given its known safety limitations, our findings do not support its direct therapeutic use, and further studies are required to validate its potential in cancer therapy.

Our findings also have a significant impact in the context of the ageing process, as peroxisomes play a vital role in maintaining cellular balance and are closely linked to ageing [65]. Since the decline of mitochondrial dysfunction contributes to ageing [66], and also peroxisomes and mitochondria interact with each other in several crucial cellular processes, their interplay is essential for cellular adaptation to ageing [3]. Therefore, peroxisomes, just like mitochondria, may play a significant role in ageing, with several studies supporting this conclusion. Activation of PPARγ, which elevates peroxisome levels, was shown to help counteract metabolic dysfunction associated with ageing. Its deacetylation also has atheroprotective properties, suppressing age-related atherosclerosis and hypercholesterolaemia [67]. Furthermore, since peroxisomes play a critical role in lipid production, they are essential for maintaining myelin and membrane integrity in the brain [68]. Consequently, dysfunction in these organelles is associated with neurodegenerative diseases and cognitive decline related to ageing [69]. These findings are in line with our data that highlighted the role of peroxisomes in protecting cells from uncontrolled growth and stemness, suggesting that improving peroxisomal function may counteract age-related decline.

Although our study did not directly investigate the relationship between ageing and cancer, our findings suggest that peroxisomal activity may represent an important point of convergence between these processes. The strong interconnection between ageing and cancer has already been well established in the literature. Accumulating evidence indicates that unrepaired DNA damage and oxidative stress disrupt cellular homeostasis, thereby contributing to both tumorigenesis and age-associated cellular decline [70,71]. Likewise, autophagy, closely linked to peroxisomal function, plays an important role in both ageing and cancer. Autophagy gene polymorphisms have been associated with age-related diseases, and several compounds have been discovered that increase autophagy, which extends lifespan and improves outcomes in age-related neurodegenerative diseases in model organisms [72]. While autophagy has a dual effect on cancer development, notably, in mouse cancer models, the loss of autophagy has been shown to reduce tumour aggressiveness, causing malignant tumours to develop into less harmful benign oncocytomas characterised by defective mitochondria [73]. Collectively, these observations highlight the potential convergence of peroxisome-associated pathways regulating ROS homeostasis, DNA integrity, and autophagy in the shared mechanisms of ageing and cancer. Future studies employing ageing-model systems will be essential to experimentally validate these potential links.

Our study has some limitations that should be addressed in future work. First, the effects of peroxisomes on stemness were examined exclusively in the MCF-7 breast cancer cell line. Although these findings provide valuable insights into the potential role of peroxisomal activity in regulating BCSC properties, they may not fully represent the heterogeneity of breast cancer biology. Future studies should include additional subtypes, such as triple-negative (MDA-MB-231) and HER2-positive (SK-BR-3) cells, to determine whether the observed effects are consistent across different molecular backgrounds [74]. Such validation would strengthen the understanding of how peroxisomal function contributes to stemness and metabolic plasticity in diverse breast cancer contexts.

Another limitation is that, although our results demonstrate a functional link between peroxisome abundance and reduced stemness through phenotypic assays, further molecular validation is required. Future work should quantify key stemness markers for BCSCs, such as SOX2, NANOG, CD44, CD24, ALDH1, and CD133 [75] and peroxisome-related genes for the biogenesis and oxidation, such as PEX3, PEX19, ACOX1, and CAT [76] to confirm these findings at the transcriptional and translational levels.

Assessing the receptor specificity of Rosiglitazone using the PPARγ inhibitor GW9662 would also help verify whether the effects are PPARγ-dependent. Additionally, evaluating EMT-related genes, migratory capacity, and chemotherapeutic responses of high- and low-peroxisome-expressing cells would further clarify whether peroxisomal signalling influences cellular plasticity and drug sensitivity. These approaches would contribute to a more comprehensive understanding of how peroxisomes regulate cancer stem cell behaviour.

In furtherance, both the quality and quantity of stem cells are critical in both processes of ageing and cancer development, suggesting a common pathway between these two phenomena [77]. Research has shown that ageing affects adult stem cell function and increases susceptibility to cancer, particularly breast cancer [78]. In addition, age-related genetic and epigenetic changes can drive the transformation of adult stem cells into CSCs [79]. In light of these findings, our results suggest that increasing peroxisomal levels, which reduce BCSCs, may be a promising solution to combat both ageing and cancer.

## 4. Materials and Methods

### 4.1. Cell Culture

MCF-7 human breast cancer cell line was purchased from the American Type Culture Collection (ATCC) and cultured in Dulbecco’s Modified Eagle Medium (DMEM; Gibco, 41966029, USA) supplemented with 10% heat-inactivated Fetal Bovine Serum (FBS; Gibco, 10082-147, USA), 1% Glutamax (Gibco, 35050-061, USA), and 1% Penicillin-Streptomycin (P/S) (Sigma-Aldrich, P0781, Poole, UK) according to the manufacturer’s instructions. Cells were grown on Nunclon Delta surface flasks (Thermo Scientific, Denmark) and maintained at 37 °C, 5% CO_2_. The cell line was passaged with 0.025% trypsin-EDTA (GIBCO, Thermo Fisher Scientific, USA) when it reached 70–80% confluency. The cell number was assessed with an automatic cell counter (BIO-RAD, TC20) by the trypan blue dye exclusion method. Cells were used at early passages (passage 10–20).

### 4.2. Plasmid and Viral Transduction

The MCF-7 cell line was transduced with pre-manufactured lentivirus carrying GFP-peroxisome or RFP-peroxisome (GenTarget Inc., San Diego, CA, USA). MCF-7 cells were seeded at 3 × 10^4^ cells per well in 6-well plates 2 days before transduction. After 3 days of incubation with the pre-prepared virus, cells were visualised under a fluorescence microscope (EVOS) to confirm successful viral transduction. Transduced cells were selected by treatment with 0.5 µg/mL puromycin until all cells in the control wells were dead.

### 4.3. Cell Sorting

GFP-peroxisome and RFP-peroxisome MCF-7 cell lines were sorted using a SONY SH800S Cell Sorter (Sony Biotechnology, Japan) to isolate the 5% of cells with the low-peroxisome content and the 5% with the high-peroxisome content as single cells. Fluorescent-tagged cells were excited using 488 nm (blue laser) and 561 nm (yellow–green laser) and selected with 405 lasers filters. The “Low” and “High” fluorescent cell sub-populations were identified by gating within the fluorescence signal. PE-A and FITC channels are chosen for RFP and GFP, respectively. Specifically, only the cells with the least (bottom 5%) and the most (top 5%) fluorescence signals were collected. Cells outside these gates were discarded during sorting based on the gate settings.

MCF-7 cells stably expressing GFP- or RFP-tagged peroxisomes were generated and subsequently sorted based on fluorescence intensity to obtain peroxisome-high and peroxisome-low subpopulations. This approach separated cells according to their endogenous peroxisome abundance, without artificial overexpression or knockdown, thereby maintaining physiologically relevant peroxisome levels within the MCF-7 population.

### 4.4. Mammosphere Formation Assay

GFP-peroxisome and RFP-peroxisome MCF-7 cell lines were seeded at a density of 5 × 10^3^ cells per well in poly-HEMA-coated 6-well plates. Poly-HEMA was prepared mixture of 1.2% poly (2-hydroxyethyl methacrylate) in 95% ethanol and 5% dH_2_O, which prevents cell attachment. The cells were sorted into 5% low-peroxisome and 5% high-peroxisome populations and seeded in Mammosphere media. The Mammosphere media consisted of serum-free DMEM/F12 without phenol red, supplemented with 0.02% Epidermal Growth Factor (EGF), 2% B27, and 1% P/S. After 7 days of incubation at 37 °C without disturbing the plates, Mammospheres (3D spheres ≥ 50 μm) were counted using a microscope.

### 4.5. Colony Assay

GFP-peroxisome and RFP-peroxisome MCF-7 cell lines were seeded at a density of 500 cells per well in 6-well plates. The cells were sorted into 5% low-peroxisome and 5% high-peroxisome populations and seeded in 10% DMEM. After incubating the cells for 14 days at 37 °C without disturbance, the formed colonies were fixed and stained for counting. The colonies were fixed with 70% ethanol at room temperature (RT) for 20 min, then stained with 0.5% crystal violet at RT for 10 min. After washing 1–2 times, the plates were left to dry overnight (O/N). The colonies were then counted using a GelCount™ (Oxford-optronix, UK) and software, with a 600-psi mask selected.

### 4.6. ATP Level Assay

GFP-peroxisome and RFP-peroxisome MCF-7 cell lines were seeded in 10% DMEM at a density of 1 × 10^4^ cells per well in glass-bottom 96-well plates. After cells were sorted into 5% low-peroxisome and 5% high-peroxisome populations, Promega CellTiter-Glo^®^ Luminescent Cell Viability Assay (Wisconsin, USA, #G9242) was applied to the cells. The plate was shaken for 15 min at RT and in the dark. The luminescence level indicating ATP level in the cells was read out by using the Varioskan™ LUX plate reader (ThermoFisher Scientific, Waltham, MA, USA).

### 4.7. Flow Cytometry Analyses of DNA Damage and Metabolic Probes

#### 4.7.1. DNA Damage

GFP-peroxisome and RFP-peroxisome cells were stained with 8-OHdG conjugated Alexa Fluor 647 antibody (Ab) (Santa Cruz Biotechnology, USA, sc-393871) to examine DNA damage. Cells were collected at a density of 5 × 10^5^ cells per tube as a cell suspension. They were fixed with 70% ice-cold ethanol O/N at −20 °C. After washing with 1× PBS, cells were stained with 8-OHdG-Alexa Ab at a 1:50 dilution for 1 h in the dark at RT. Without additional washing, both stained and control cells were analysed using the ATTUNE NxT Flow Cytometry (ThermoFisher Scientific, Waltham, MA, USA).

#### 4.7.2. Mitochondrial Superoxide Detection

GFP-peroxisome and RFP-peroxisome cells were examined with MtSOX Deep Red (Dojindo Laboratories, Japan, MT14-10) to determine the level of mitochondrial-superoxidase production. Cells were collected at a density of 300,000 cells per tube as a cell suspension. Live cells were incubated with 10 µM of MtSOX for 30 min at 37 °C in the dark and washed with 1× PBS. Subsequently, stained and control cells were analysed by flow cytometry.

#### 4.7.3. Lipid Peroxide Detection

RFP-peroxisome cells were examined with Liperfluo (Dojindo Laboratories, Japan, L248-10) to analyse lipid peroxidation level. Cells were collected at a density of 3 × 10^5^ cells per tube as a cell suspension. Live cells were incubated with 10 µM/l of Liperfluo for 30 min at 37 °C in the dark and washed with 1× PBS. Subsequently, stained and control cells were analysed by flow cytometry.

#### 4.7.4. Mitochondrial Lipid Peroxide Detection

RFP-peroxisome cells were examined with MitoPeDPP (Dojindo Laboratories, Japan, M466-10) to analyse mitochondrial lipid peroxidation level. The cells were collected as a suspension, with each tube containing a density of 3 × 10^5^ cells. Live cells were incubated with 2 µml/l of MitoPeDPP for 30 min at 37 °C in the dark and washed with 1× PBS. Subsequently, stained and control cells were analysed by flow cytometry.

#### 4.7.5. Autophagy

GFP-peroxisome and RFP-peroxisome cells were prepared as a suspension at 3 × 10^5^ cells per tube and stained with DAPRED (Dojindo Laboratories D677-10) and DAPGREEN (Dojindo Laboratories, Japan, D676), respectively, to see the autophagy level. RFP-peroxisome cells were incubated with 0.2 µmol/l of DAPGREEN for 30 min at 37 °C in the dark. GFP-peroxisome cells were incubated with 0.4 µmol/l of DAPRED for 30 min at 37 °C in the dark. Following washing steps with 1× PBS, stained and unstained cells were examined using flow cytometry.

#### 4.7.6. Intracellular Fe^+2^ Level

GFP-peroxisome cells were prepared as a suspension at 5 × 10^5^ cells per tube. The live cells were incubated with 10 µM of RhoNox-1 (MedChemExpress, 10 mM, USA, HY-D1533) for 30 min at RT in the dark. After the cells were washed with 1× PBS, stained and unstained cells were analysed by using flow cytometry.

#### 4.7.7. Cell Cycle Analyses

RFP-peroxisome cells were sorted at 2.5 × 10^5^ cells per tube as 5% low-peroxisome and 5% high-peroxisome populations. Subsequently, the cells were fixed with 70% ice-cold ethanol O/N at −20 °C. After washing with 1× PBS, the cells were incubated with Muse^®^ Cell Cycle Kit (Luminex, Massachusetts, USA, #MCH100106) for 30 min at RT in the dark. Without additional washing, both stained and unstained cells were analysed using flow cytometry.

#### 4.7.8. Flow Cytometry

For GFP, Liperfluo, and DAPGREEN detection, the BL1-A channel was used. For RFP and RhoNox-1 detection, the YL1-A channel was used. For MitSOX and 8-OHdG detection, the RL1-A channel was used. For 8-OHdG detection, the RL1-A channel was used. For MitoPeDPP detection, the VL2 channel was used. For DAPRED, the YL3 channel was used. For the cell cycle, the BL2-A channel was used. No compensation was required as fluorochromes were selected to avoid spectral overlap. When the cells were not sorted as low and high populations, the lowest 5% and highest 5% of the gated cell populations were identified and analysed using FlowJo software v10.

### 4.8. Seahorse XFe96 Metabolic Flux Analysis

Real-time OCRs and ECARs were measured using the Seahorse Extracellular Flux (XFe96) analyzer (Seahorse Bioscience, North Billerica, MA, USA). GFP-peroxisome and RFP-peroxisome cells were sorted into 5% low and 5% high-peroxisome content groups. After sorting, 5 × 10^4^ cells per well were seeded into XFe96 well cell culture plates and incubated for 24 h to allow cell attachment. Following the 24 h incubation, the cells were washed with pre-warmed XF assay media. For ECAR measurements, the cells were maintained in XF assay media. For OCR measurements, the XF assay media was supplemented with 10 mM glucose, 2 mM pyruvate, and 2 mM L-glutamine, adjusted to pH 7.4. The cells were then maintained in 175 μL of XF assay media per well for 1 h at 37 °C in a non-CO_2_ incubator. During this incubation period, 25 μL of ECAR and OCR solutions were prepared and loaded into the injection ports of the XFe96 sensor cartridge, which had previously been incubated with Agilent Seahorse XF calibrant for at least 1 h at 37 °C in a non-CO_2_ incubator. ECAR media were prepared by adding 80 mM glucose, 9 μM oligomycin, and 1 M 2-deoxyglucose to XF assay media. OCR media were prepared by adding 10 μM oligomycin, 9 μM FCCP, 10 μM rotenone, and 10 μM antimycin A to XF assay media. Measurements were normalised to Hoechst 33342 content. Data sets were analysed using XFe96 software and GraphPad Prism software, employing unpaired Student’s *t*-tests. All experiments were performed in triplicate and repeated three times independently.

### 4.9. Rosiglitazone Treatment

MCF-7 cells were seeded at a density of 150,000 cells per well in a 6-well plate and incubated for 24 h. Rosiglitazone (ChemScene, New Jersey, USA, CS-1088) was prepared by dissolving it in DMSO. The cells were then treated with Rosiglitazone at various concentrations (1 µM, 10 µM, 50 µM, 75 µM, and 100 µM) along with a DMSO control for 3 days. After treatment, the cells were collected and examined for lipid peroxidation using Liperfluo as previously described.

For the Mammosphere assay, Rosiglitazone at different concentrations was prepared and mixed into the Mammosphere media in a 6-well plate. MCF-7 cells were seeded at 3000 cells per well using a 25 G needle and a 1 mL syringe to ensure single-cell suspension, then incubated for 5 days.

For the colony assay, MCF-7 cells were seeded at 500 cells per well using a 25 G gauge and a 1 mL syringe to ensure single-cell suspension, then incubated for 2 weeks.

### 4.10. Statistical Analyses

All analyses were performed using GraphPad Prism 10. Data are represented as mean ± SD where indicated. All experiments were independently conducted at least three times as biological replicates (N), with a minimum of two technical replicates (*n*) for each experimental condition tested. Statistically significant differences were determined using the Student’s unpaired *t*-test, with *p* < 0.05 considered significant.

## 5. Conclusions

Peroxisomes are often overlooked as potential targets for cancer and ageing therapies. In this study, we explored the effects of elevated peroxisome levels on cancer stemness and metabolism in breast cancer. High-peroxisome levels were achieved by transducing MCF-7 cells with GFP- or RFP-tagged peroxisomes or by treating MCF-7 cells with the PPARγ agonist, Rosiglitazone. Our findings indicate that high levels of peroxisomes can significantly reduce the stemness of BCSCs. This reduction in stemness may be linked to increased ROS levels, potentially triggering autophagy, DNA damage, and ferroptosis. Our study thus outlined increasing peroxisomes, most importantly via the use of PPARγ agonists, as a therapeutic strategy against cancer development and invasiveness. However, further research is needed to confirm these mechanisms and draw definitive conclusions.

## Data Availability

All data are available upon request.

## Abbreviations

The following abbreviations are used in this manuscript:

ROS	Reactive oxygen species
ER	Endoplasmic reticulum
ATCC	American Type Culture Collection
DMEM	Dulbecco’s Modified Eagle Medium
P/S	Penicillin–streptomycin
Poly-HEMA	Poly (2-hydroxyethyl methacrylate)
EGF	Epidermal Growth Factor
RT	Room temperature
O/N	Overnight
OCRs	Oxygen consumption rates
ECARs	Extracellular acidification rates
PI	Propidium iodide
BCSCs	Breast cancer stem cells
CSCs	Cancer stem cells
FCCP	Carbonyl cyanide-p-trifluoromethoxy phenylhydrazone
2-DG	2-deoxyglucose
T2DM	Type 2 diabetes mellitus
PPARγ	Peroxisome proliferator-activated receptor gamma
